# Uptake of Technology for Neurorehabilitation in Clinical Practice: A Scoping Review

**DOI:** 10.1093/ptj/pzad140

**Published:** 2023-10-19

**Authors:** Margit Alt Murphy, Sujata Pradhan, Mindy F Levin, Nicola J Hancock

**Affiliations:** Department of Clinical Neuroscience, Rehabilitation Medicine, Institute of Neuroscience and Physiology, Sahlgrenska Academy, University of Gothenburg, Gothenburg, Sweden; Department of Occupational Therapy and Physiotherapy, Sahlgrenska University Hospital, Gothenburg, Sweden; Division of Physical Therapy, Department of Rehabilitation Medicine, University of Washington, Seattle, Washington, USA; School of Physical and Occupational Therapy, Faculty of Medicine and Health Sciences, McGill University, Montreal, Quebec, Canada; Centre for Interdisciplinary Research in Rehabilitation of Greater Montreal (CRIR), Montreal, Quebec, Canada; School of Health Sciences, Faculty of Medicine and Health Sciences, University of East Anglia, Norwich, UK

**Keywords:** Neurological, Rehabilitation, Neurological Conditions, Rehabilitation, Scoping, Technology, Translation, Uptake

## Abstract

**Objective:**

Technology-based interventions offer many opportunities to enhance neurorehabilitation, with associated research activity gathering pace. Despite this fact, translation for use in clinical practice has lagged research innovation. An overview of the current “state of play” regarding the extent of clinical uptake and factors that might influence use of technologies is required. This scoping review explored the uptake of technologies as neurorehabilitation interventions in clinical practice and factors that are reported to influence their uptake.

**Methods:**

This systematic scoping review was conducted with narrative synthesis and evidence mapping. Studies of any design reporting uptake or implementation of technology (wearable devices, virtual reality, robotics, and exergaming) for movement neurorehabilitation after stroke and other neurological conditions were sought via a formal search strategy in MEDLINE (Ovid), CINAHL, AMED, and Embase. Full-text screening and data extraction were completed independently by 2 reviewers.

**Results:**

Of 609 studies returned, 25 studies were included after title, abstract, and full-text screening. Studies investigated a range of technologies at various stages of development. Only 4 of the included studies explored the sustained use of technology in practice. The following 5 themes representing experiences of technology use emerged: perceived usefulness, technology design, social interaction, integration with services, and suggested improvements to enhance uptake.

**Conclusion:**

Reporting of uptake and use of neurorehabilitation technologies in clinical practice is limited. The synthesis provided comprehensive knowledge of barriers to and facilitators of uptake to be considered in future protocols, including a steep learning curve required to engage with technology, a need for a supportive organizational culture, and a need for user involvement in both design and development.

**Impact:**

This scoping review has provided indicators from current evidence of important factors to consider in the planning of research into and clinical implementation of technologies for neurorehabilitation. It serves to support an evidence-based, user-centered platform for improved research on and translation of technologies in neurorehabilitation clinical practice.

## Introduction

Technology-based interventions, such as robotics, wearable devices, virtual reality systems, and other exergaming devices, offer opportunities to enhance neurorehabilitation in several ways. These include, but are not limited to, increasing intensity and repetitions of task-oriented therapy,^1^ provision of support for self-management and personalization of therapy,^1–3^ and enhancing interest and participation in rehabilitation activity.^4^ It is not surprising that there has been a proliferation of publications at the intersection of technology and neurorehabilitation in recent years^5^ as both technological advances and the associated research activity gather pace.

In contrast to these advances, translation to and uptake of research findings into clinical practice lag behind research innovation^6^ even in neurorehabilitation,^7^ sometimes by decades. This lack of clinical implementation presents an urgent problem, as opportunities to enhance standards of individual patient care and service delivery may be lost to health systems at a time when they face unprecedented pressures and demands alongside increasingly constrained resources. Comprehending this translational gap necessitates a broad understanding of the current “state of play” regarding the extent of clinical uptake and the factors that might influence the use of technologies in practice.

A variety of studies have sought to understand some of the challenges faced. Reported influences on adoption include the nature of the devices themselves, the patient–clinician relationship, management support, and the health system/context in which the technologies are being used.^3^^,^^8^ The importance of the rehabilitation clinician in helping people to make the right choices about the right technology to meet their needs has been recognized.^9^ Collaboration between health care professionals, developers, and intended users of devices is considered to be of value to clinical applicability.^1^^,^^5^ Such a collaboration may lead to a greater acceptability and uptake of technology in clinical practice. However, little is known about the extent of clinical uptake of technological interventions.

Although individual studies have demonstrated some of the challenges inherent in clinical translation of technological advances as neurorehabilitation interventions, a broad synthesis of the current evidence is required to further develop the understanding about uptake and to clarify knowledge gaps. Such a synthesis is possible using scoping review methodology and might provide a clear direction from current literature to inform the development of future user-centered protocols for research and practice.

## Objective

In line with recommendations on conducting a systematic scoping review,^10^^,^^11^ the overarching broad objective of this review was to collate knowledge about the uptake of technology in neurorehabilitation practice. The objective was explored using the following review questions: Primarily, what is the uptake of technologies (not including neuromodulation) as neurorehabilitation interventions in clinical neurorehabilitation practice in adults? Secondarily, what factors are reported to influence the uptake of those technological interventions in adult neurorehabilitation?

The driver for this review was further generated from an international online seminar hosted by the International Neurological Physical Therapy Association (INPA, a subgroup of the World Physiotherapy Association) in December 2021, centered on technologies for neurorehabilitation practice, attended by delegates representing research and practice from many countries.

## Methods

### Study Design

We carried out a scoping review and synthesis with evidence mapping. This review method was considered as the most appropriate to meet our aims, as it is a recommended tool for reconnaissance of evidence of a heterogeneous nature in a complex field for summarizing and mapping existing evidence and for consideration of recommendations for future research,^10^^,^^11^ including generation of questions for future systematic reviews. As scoping reviews do not usually include critical appraisal of methodological qualities and are carried out in shorter timelines than traditional systematic reviews, there is a potential for the introduction of biases. To minimize this, our review was carried out systematically, with steps to assure quality and reduce error, such as a rigorous search process, independent screening of full texts, and data extraction according to predetermined criteria. Our report is in accordance with the published recommendations.^10^

### Search Strategy and Sources

A formal search strategy was developed by the authors and the Faculty of Medicine and Health Sciences Librarian at the University of East Anglia, United Kingdom, to capture terms relevant to the research question (Suppl. Material I). All authors have extensive experience of both neurorehabilitation practice (≥10 years each) and research (≥15 years each).

Technology for neurorehabilitation presents a heterogeneous field for scoping work, and so key decisions on keywords and index terms were discussed during the development of the strategy and criteria for the review to ensure a breadth of relevant papers were included, but with sufficient specificity. This included definitions of the types of technology to be included. The authorship team decided that “stroke” should be included as a named condition as there are a prevalent number of publications in this area but that neurorehabilitation should be included as an overarching term. Databases searched were MEDLINE (Ovid), Embase, CINAHL, and AMED.

Searches were refined and final searches for studies reported here were carried out on June 7, 2022 (MEDLINE, Embase) and on September 11, 2022 (CINAHL, AMED—a later date because the initial quality checks led to a requirement to enhance the subject headings for these databases).

### Framework

A population, concept, and context framework was used^10^ to underpin the review and to guide data extraction and mapping. Using this framework, we considered the population (eg, characteristics of “participants,” defined as clinical users of technology, including people with neurorehabilitation needs and clinicians); the concept (eg, the phenomena of interest, such as uptake of neurorehabilitation technology); and the context (eg, but not limited to, geographical location, setting, type of study, participants, and length of evaluation).

### Study Selection and Data Extraction

The inclusion criteria were as follows: studies reporting the uptake/implementation or factors influencing the uptake/implementation of technology for movement neurorehabilitation after stroke and other neurological conditions**;** technology for movement neurorehabilitation (wearable devices, virtual reality, robotics, and exergaming); primary research studies using quantitative or qualitative outcomes to explore uptake; clinicians or people with neurorehabilitation needs; adults who were 18 years old or older; and articles published in the English language.

The exclusion criteria were as follows: studies reporting the evaluation and uptake of telerehabilitation delivery of services and interventions; studies reporting the evaluation and uptake of artificial intelligence or neuromodulation devices; studies reporting the evaluation and uptake of communication devices; protocols and proceedings; and review articles.

The authorship team worked collaboratively through the review process and met regularly in the decision-making stages. Following the removal of duplicates, returned studies were divided equally and randomly between the team, and titles and abstracts were checked against the predetermined criteria for inclusion. A 10% sample from each author was then cross-checked by a different team member. The disagreements were discussed at the next review team meeting.

A database of studies for full-text review was then created and divided between the team for independent review for inclusion. The sample of papers was divided in half, and 2 authors independently screened one-half each (N.J.H. and S.P.; M.A.M. and M.F.L.). A meeting was then held to review the decisions made and to resolve any disagreements.

A data extraction pro forma was developed to capture the essential information for mapping by 2 authors (M.F.L. and S.P.) and this was agreed by the team (Suppl. Material II). Data extraction was divided between the team members. For the qualitative synthesis of user perspectives, the results of the qualitative individual or focus group interviews and written vignettes were screened by 2 researchers (N.J.H. and M.A.M.). The reported themes and subthemes were extracted and tabulated to identify the similarities and commonalities across the studies agreed between M.A.M. and N.J.H.

### Synthesis and Presentation of Findings

The plan for results presentation was refined throughout the review process and a narrative synthesis was supported by “evidence mapping.” This is a recognized technique to identify future research needs following a systematic search by using formats, such as figures and tables, to depict key findings.^11^

## Results

**Figure 1:**
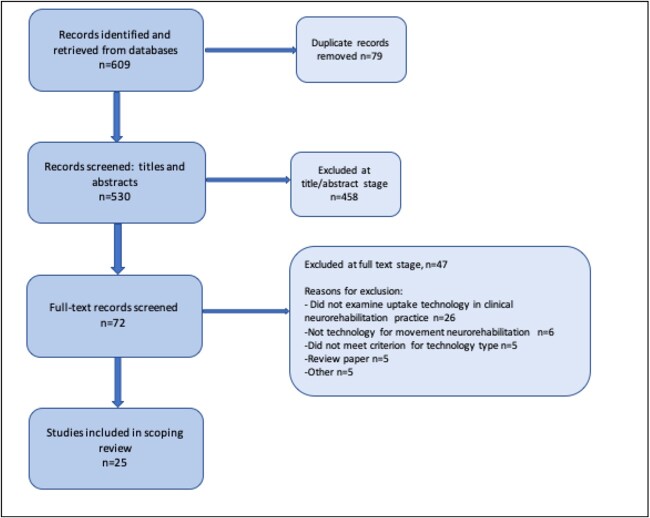
Flowchart illustrating the study identification, retrieval, and selection process.

Following a systematic search and review process (Fig. 1), 25 studies were included in the review. Based on our primary objective to review the uptake of technologies in clinical neurorehabilitation practice, we found only 4 studies that investigated the uptake or factors influencing the uptake after sustained use (5 months–3 years) of technology in clinical practice.^8^^,^^9^^,^^12^^,^^13^ There were no studies resulting from our search that directly investigated the extent of uptake in a naturalistic, real-world clinical environment.

### Summary of the Included Studies

**Figure 2:**
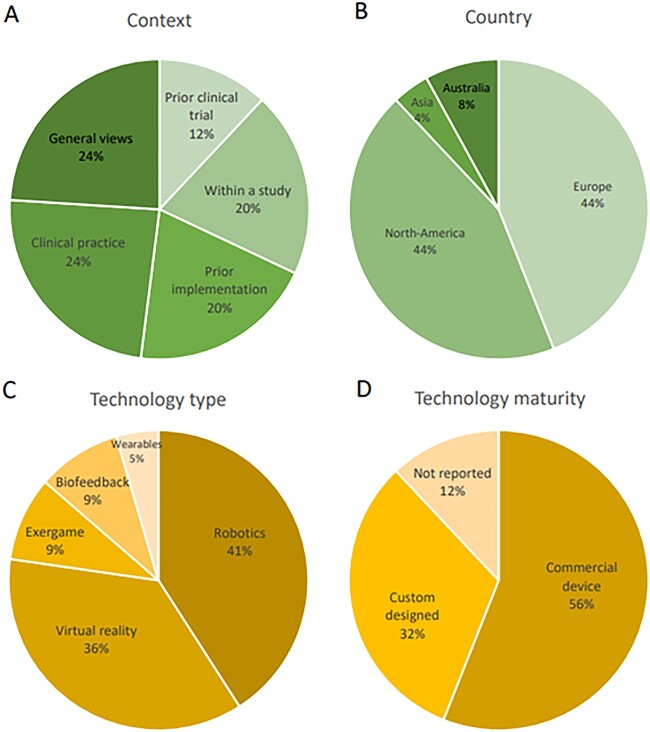
Characteristics of the studies according to A) context B) study country C) technology type and D) technology maturity. Wearables = wearable devices.

The main extracted characteristics of the included 25 studies are shown in Table 1. Studies had investigated the uptake in neurorehabilitation and/or user perspectives of technology either prior to a randomized controlled trial (*n* = 3) or after use within a clinical study (*n* = 5), prior to planned implementation (*n* = 5) or when the technology was already integrated into clinical practice (*n* = 6). The remaining 6 studies explored more general views of the clinicians on the use of rehabilitation technology in clinical practice (Fig. 2). The main user groups were clinicians and people with a neurological condition, and 6 studies incorporated both groups. With the exception of the 3 large surveys,^14–16^ which included a total of 618 respondents (495 health care professionals and 123 patients and carers), the sample sizes in all other studies were small, ranging from 3 to 27 participants (mean = 9.6 [SD = 5.0] participants), with 285 participants in total. Of these, 143 were health care professionals (physical therapists and occupational therapists), 7 were other professionals (engineers, administrators), and 135 were people with neurological conditions (124 with stroke, 7 with spinal cord injury, 3 with traumatic brain injury, and 1 with cerebral palsy). In studies that investigated technology for people with stroke, 74% of people were in the subacute stage of stroke recovery, and 26% were in the chronic stage of stroke recovery.

**Table 1:** Characteristics of the 25 Included Studies*^a^*

Study	Population	Type of Study	Focus of Interest	Type of Technology	Setting
Prior to an RCT					
Ballinger et al^31^ (2016)	16 clinicians: 9 occupational therapists, 5 physical therapists, 2 orthotists	Qualitative interview	Clinicians’ perspectives and expectations prior to an RCT	Visualization of biomechanics Custom-designed	Hospital and community stroke rehabilitation
Demers et al^23^ (2019)	7 people with subacute/chronic stroke; 9 occupational therapists	Mixed methods: qualitative interview (stroke), focus group (occupational therapist), survey (stroke)	Patients’ and clinicians’ perceptions (satisfaction, adverse effects, usefulness, and safety) prior to an RCT	VR gaming Custom-designed	Stroke rehabilitation center
Hoermann et al^17^ (2017)	Part 1: 5 people with subacute stroke; 3 occupational therapists Part 2: 12 people with subacute stroke	Survey (system evaluation, user experience, feasibility, and adherence)	Patients’ responses after use (3–13 sessions) prior to an RCT	UL VR, augmented reflection technology Custom-designed	Stroke rehabilitation center
After use within a study					
Louie et al^18^ (2021)	14 people with subacute stroke; 6 physical therapists	Qualitative interview	Patients’ and clinicians’ experience and acceptability after use within an RCT	LL exoskeleton (EksoGT)	Hospital stroke rehabilitation
Gustavsson et al^19^ (2021)	7 people with chronic stroke	Qualitative interview	Patient’s experience after use within a single-subject experimental study of 10 wk of use	Fully immersive VR for UL	Research laboratory
Standen et al^20^ (2015)	13 people with subacute/chronic stroke	Mixed methods: qualitative interview, clinical outcomes	Patients’ use time, feasibility, and uptake after an 8-wk feasibility RCT	VR glove gaming (sensors, Kinect) Custom-designed	Home setting with weekly support
Valdés et al^21^ (2018)	10 people with chronic stroke/TBI/CP; 12 clinicians: 3 occupational therapists, 2 physical therapists, 1 administrator, 6 engineers	Qualitative interview (notes and quotes), documentation, group discussions	Patients’, clinicians’, and technology developers’ experience of and factors influencing use within a pilot clinical trial	UL VR gaming (FEATHERS) Custom-designed	Home setting with weekly support
Lee et al^30^ (2016)	8 people with subacute stroke	Mixed methods: ratings, documentation, qualitative interview	Patients’ perceived levels of difficulty, training intensity, assistance needed, pain, and enjoyment during and after intervention within an explorative mixed-methods study	VR (Kinect) (workout/gaming) Custom-designed	Hospital (acute stroke)
Prior to implementation					
Flynn et al^24^ (2019)	12 clinicians: 6 physical therapists, 6 occupational therapists	Focus groups	Clinicians’ perceptions about barriers/enablers prior to implementation as the clinical routine	UL robotic therapy (InMotion 2)	Hospital stroke rehabilitation
Feldner et al^29^ (2019)	22 clinicians: 13 occupational therapists, 7 physical therapists, 2 physiatrists	Qualitative interview, focus groups	Clinicians’ perceptions (values, benefits, and drawbacks) prior to implementation in clinical practice	Surface EMG	Neurorehabilitation centers
Cherry et al^32^ (2017)	10 people with chronic stroke	Observation, qualitative interviews	Patients’ perceptions on effectiveness of, benefits of, and barriers to use within a pilot implementation study	Robotic therapy with gaming (Kinetic Muscles, Hand Mentor, and Foot Mentor)	Home setting (rural)
Levac et al^8^ (2016)	11 clinicians: 6 physical therapists, 5 occupational therapists	Focus groups, ratings of use	Clinicians’ perspectives on intervention knowledge translation, usability of VR within initial introduction, and sustained use (6 mo) of the system in clinical practice	VR system (IREX GestureTek)	Stroke rehabilitation center
Tetteroo 2014^36^	2 occupational therapists, 1 physical therapist (no data on patients)	Mixed methods: qualitative interview, questionnaires	Clinicians’ perception of self-efficacy, acceptance, and credibility/expectations after 1–2 wk of use within an implementation study in clinical practice	Exercise tablet for UL rehabilitation (TagTrainer) Custom-designed	Hospital stroke rehabilitation
After use within clinical practice					
Swank et al^13^ (2020)	16 people with subacute stroke, 7 people with subacute SCI; 4 physical therapists	Mixed methods: focus groups (physical therapist), survey (stroke)	Patients’ and clinicians’ experience on use, satisfaction, and feasibility within a convenient-sample feasibility study over 6 mo of use in clinical settings	LL exoskeleton (EksoGT)	Hospital stroke rehabilitation
Schmid et al^25^ (2016)	9 clinicians: 6 physical therapists, 3 occupational therapists	Focus group	Clinicians’ experience of and expectations about facilitators of and barriers to implementation after use in clinical practice	VR UL (YouGrabber) Custom-designed	Neurological rehabilitation centers
Nguyen et al^9^ (2019)	10 clinicians: 6 occupational therapists, 4 physical therapists	Qualitative interview	Clinicians’ perceptions (about acceptance, expectations, and facilitators) after some experience during 12 mo within the clinical routine	Exergame room use (Jintronix and Meditouch)	Stroke rehabilitation hospital
Stephenson and Stephens^33^ (2018)	6 physical therapists	Qualitative interview	Clinicians’ experience on use after some experience during the last 5–12 mo with robotic training within the clinical routine	UL robotic therapy (InMotion 2)	Stroke rehabilitation center
Høyer et al^22^ (2022)	26 people with subacute stroke	Survey (Likert scale)	Patients’ experience of training, satisfaction, and usefulness after 3 wk of training within an exploratory study in a clinical practice environment	LL exoskeleton (EksoGT)	Hospital stroke rehabilitation
Swank et al^12^ (2020)	10 physical therapists	Mixed methods: focus groups, survey	Clinicians’ experience on use, satisfaction, feasibility, and sustainability over 3-y of use in clinical practice	LL exoskeleton (EksoGT)	Stroke rehabilitation center
General views					
Hughes et al^14^ (2014)	415 respondents: 99 with chronic stroke, 24 carers, 269 clinicians	Mixed methods: codesign survey development, focus groups, survey output	Patients’, carers’, and clinicians’ familiarity with, use of, and perceptions on UL technology in general	All UL technologies	Exhibition on assistive technologies
Li et al^15^ (2021)	100 clinicians: 62 physical therapists, 35 occupational therapists	Mixed methods: survey with open and closed questions	Clinicians’ experience and views of use; to understand the reasons for low levels of implementation	Rehabilitation robots	All clinical settings
Read et al^26^ (2020)	3 physical therapists	Qualitative interview	Clinicians’ views on how the use of technology (within research) has affected their clinical work	LL exoskeleton (EksoGT)	Neurological rehabilitation
Celian et al^28^ (2021)	5 clinicians (occupational therapists and physical therapists)	Qualitative analysis of written vignettes	Clinicians’ real-time decision-making process on the use of technology in treatment	Rehabilitation technology	Rehabilitation research hospital
Braakhuis et al^16^ (2021)	103 physical therapists	Survey with open and closed questions	Clinicians’ views on potential barriers to applications in clinical practice	Wearable activity monitoring	Stroke rehabilitation
Bower et al^27^ (2021)	9 clinicians: 9 physical therapists, 9 occupational therapists	Focus groups	Clinicians’ views on using technologies for assessment and treatment	Electronic technologies	Neurological rehabilitation

^a^ CP = cerebral palsy; EMG = electromyography; LL = lower limb; RCT = randomized controlled trial; SCI = spinal cord injury; TBI = traumatic brain injury; UL = upper limb; VR = virtual reality.

Fourteen studies adopted a qualitative approach, analyzing individual interviews, written vignettes, or focus group interviews; 8 studies had a mixed-methods design, and 3 studies used surveys as the main outcome for evaluation of user perspectives. All studies were conducted at high-income countries, most commonly in Europe or North America (Fig. 2). Most studies were conducted at a hospital or rehabilitation center setting; only 3 were conducted at a home environment. All interventions conducted at hospital or rehabilitation centers were supervised by the therapists, while the home interventions were semisupervised with a weekly support.

The studies included a range of the technologies, but robotic devices and virtual reality were the most investigated (Fig. 2). Seven studies had a specific focus on upper limb, 5 on lower limb and 8 included both. More than half of the studies explored the use and uptake of commercial products, while about 30% evaluated technologies at various stages of prototype development (Fig. 2).

### Synthesis of Evidence on Uptake of Technologies

Sixteen studies provided information on the targeted use time, actual use time, adverse events, and reasons for dropouts or missed sessions (Tab. 2). Six studies^17–22^ specified a recommended time of use for the technology in their protocol. Even when some participants reached the recommended time of use, the average number of sessions at the group level was approximately half of the targeted number of sessions.^17–21^

**Table 2:** Targeted and Actual Use Times, Adherence, Sustainability, and Uptake Where Reported in Included Studies (*n* = 16)*^a^*

Study	Targeted Use Time	Actual Use Time	Adverse Effect and Reasons for Missed Sessions or Dropout	Sustained Use in Clinical Practice	Type of Technology
Prior to an RCT					
Demers et al^23^ (2019)	Single session of 20–45 min	Single session, on average, 4.7 games, 31 repetitions, in 5 min with affected arm	57% reported temporary eye tiredness	N/A	VR gaming
Hoermann et al^17^ (2017)	Patients’ preference, but 20 sessions of 30 min recommended	On average, 9 sessions/patient; only 2 participants completed all scheduled sessions (9 and 12 sessions)	Missed sessions due to not feeling well, patient was not available	Not reported	UL VR (augmented reflection technology)
After use within a study					
Louie et al^18^ (2021)	45 min, 3 times/wk, over 8 wk (24 sessions)	On average, 12.5 (SD = 4.1) sessions/patient	Fatigue after training	Not reported	LL exoskeleton (EksoGT)
Gustavsson et al^19^ (2021)	30–45 min, 3 times/wk, over 10 wk (30 sessions)	On average, 16.2 (range = 9–27) sessions/patient; mean = 587 (range = 198–915) min/session	Missed sessions due to transport, busy schedule	N/A	Fully immersive VR for UL
Standen et al^20^ (2015)	Patients’ preference, but 20 min, 3 times/wk, over 8 wk recommended (24 sessions)	On average, completed 46% (range = 10%–100%) of sessions, with a session duration of 17.5% (range = 1.5%–70%) of the recommended use; 1 patient did not reach 8 wk of training time	Reasons for lack of adherence: health problems, competing commitments, technical issues, fatigue, dependence on assistance	N/A	VR glove gaming
Valdés et al^21^ (2018)	Asked to use 30 min, 5 d/wk, over 8 wk (weekly goal of 150 min/wk)	Of 10 patients, 5 completed the weekly goal for 8 wk, 3 completed it for 4 or 5 wk, and 2 completed it for 0 wk	Reasons for not completing: technology issues, time commitment, compensatory movements	Not reported	UL VR gaming (FEATHERS)
Lee et al^30^ (2016)	Same as actual use time	20–30 min, 3 d/wk, for a total of 5–8 sessions	Not reported	Not reported	VR (Kinect) (workout/gaming)
Prior to implementation					
Cherry et al^32^ (2017)	Recommended to use 2 h/d, over 3 mo	Not reported	Not reported	Not reported	Robotic therapy (Hand Mentor and Foot Mentor)
Levac et al^8^ (2016)	Knowledge translation program for 6 mo and sustained use over 6 mo	Of 11 therapists, 5 reached the target recruitment of 4 patients; 5/9 agreed to sustained use, but only 2 used the technology 1 or 2 times	Reasons for not using: lack of time, location, technical issues, lack of appropriate patients	Sustained use >6 mo after intervention was minimal	VR system (IREX)
Tetteroo^36^ (2014)	1-wk of introduction, 2 wk of use	34 therapy sessions; 20 were group sessions; 4 therapists created 20 new exercises	Not reported	Not reported	Exercise tablet for UL rehabilitation (TagTrainer)
After use within clinical practice					
Swank et al^13^ (2020)	Use over 6 mo	On average, 4.5 sessions/patient (3–13 min of walking time, 100–400 steps/session)	Reasons for limited uptake: resources, management, implementation process No adverse events	Feasibility improved over 6 mo but took longer than anticipated	LL exoskeleton (EksoGT)
Schmid et al^25^ (2016)	Not reported	Therapists reported that, on average, 15.5 (range = 2–60) patients had used the device	Not reported	Not reported	VR UL (YouGrabber)
Nguyen et al^9^ (2019)	1 y after implementation program	All 10 participating clinicians had referred patients to the exergame room	Not reported	Facilitators and barriers were identified	Exergame room use (Jintronix, Meditouch)
Stephenson and Stephens^33^ (2018)	Not reported	Therapist with experience using the technology (5–12 mo, last use varied from same week to 1 y before)	Not reported	Not reported	UL robotic therapy (InMotion 2)
Høyer et al^22^ (2022)	60 min, 2 or 3 times/wk, over 3 wk (6–9 sessions)	Median of 7 sessions/patient over 19.5 d (17–22 min of walking time, 590–810 steps/session)	Mean recruitment rate of 10%; 6 patients dropped out (motivation, discomfort/pain); few adverse events were related to discomfort; 7% of sessions were canceled	Not reported	LL exoskeleton (EksoGT)
Swank et al^12^ (2020)	3 y of use	Mean of 1.85 y of experience with use; most had performed >50 sessions, 1–5 times/wk	3 adverse events in a total of 186 patients	Perceived experience of sustained use	LL exoskeleton (EksoGT)

^a^ LL = lower limb; N/A = not applicable; RCT = randomized controlled trial; UL = upper limb; VR = virtual reality.

The most common reasons for missing sessions were health problems, competing commitments, technical issues, fatigue, dependence on assistance, patient not available, lack of time, or lack of appropriate patients. Three studies^16^^,^^22^^,^^23^ reported adverse effects, such as tiredness and discomfort (virtual reality and lower limb exoskeleton) in a few cases; 1 study using lower limb exoskeleton did not specify the type of adverse effect^12^ and another study using lower limb exoskeleton reported no adverse effects.^13^

Only 4 studies^8^^,^^9^^,^^12^^,^^13^ explored the sustained use of technology (virtual reality, lower limb exoskeleton, and exergame room use) after the initial program. One study^13^ reported improved feasibility after using lower limb exoskeleton in clinical practice over 6 months and also concluded that the time needed for implementation was longer than anticipated. One study^8^ reported that, despite gained knowledge, positive attitude, and intention for continuous use, only 2 of 5 clinicians used the technology (virtual reality system) in a few sessions after the implementation program. Two remaining studies^9^^,^^12^ reported qualitative data from interviews on the perceived experiences of use among the clinicians 1 year and 3 years after sustained use. The identified factors that influenced the uptake and use were similar in all stages of implementation and are reported together with all studies below.

### Qualitative Synthesis of User Perspectives

**Figure 3:**
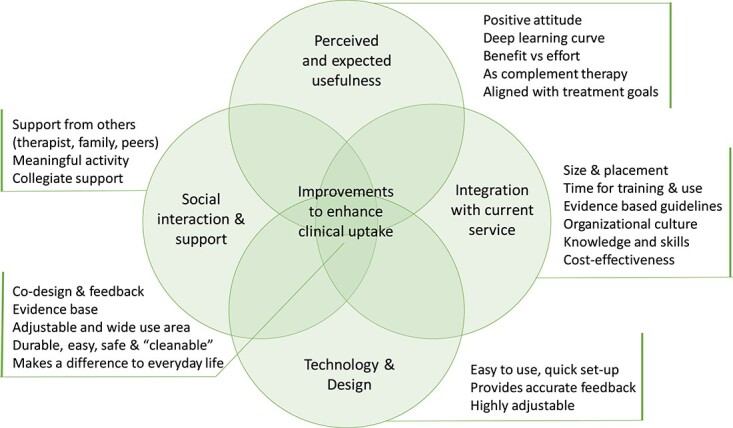
Synthesis of user perspectives on factors influencing the uptake and implementation of technology in neurorehabilitation clinical practice.

Exploration of themes and subthemes from the included papers had several overlapping elements independent of the stage of implementation or technology type. Therefore, the synthesis was carried out across all included studies. Five overarching themes emerged representing the perceptions and experiences of the use of technology in neurorehabilitation. These were: perceived and expected usefulness, technology design, social interaction and support, integration with current service, and improvements to enhance the clinical uptake. Figure 3 summarizes the synthesis via the themes and their interactions.

### Perceived and Expected Usefulness

Clinicians and people with neurological conditions were positive and optimistic about their experiences with technologies and found them to be exciting and motivating,^8^^,^^13^^,^^18^^,^^19^^,^^24–27^ but they described an initial steep learning curve^18^^,^^26^ and a need for continuous training in the use of technology.^8^^,^^12^^,^^15^^,^^21^^,^^24^^,^^26^^,^^28^ Robotic devices were seen as potential tools to increase the amount of therapy and to broaden the range of therapy options available.^15^^,^^26^^,^^28^ Clinicians reflected that robotic devices will not suit everyone^13^^,^^26^ and that settings with longer rehabilitation times enabled people to become familiar with the features, and clinical use, of technologies.^13^ There was recognition that perceived usefulness and benefit need to be higher than the cost and effort and that progress and effects on daily activities should be apparent.^13^^,^^18^^,^^26^^,^^28–30^ Training-related exertion and fatigue influenced perceptions of usefulness of exoskeletons, and therapists were concerned about the expected benefits compared to the effort expenditure to carry out training.^18^ People with stroke and spinal cord injury perceived greater improvements and benefits of the use of exoskeleton training than therapists.^13^^,^^26^ Clinicians emphasized a need for technologies to be integrated with clinical decision-making and therapy goals.^8^^,^^12^^,^^13^^,^^27^ Technologies were considered to work as a complement to existing therapy, particularly where they enabled objective measurement and standardization, and to increase people’s involvement in their therapy, supporting a sense of control for people using them.^13^^,^^15^^,^^19^^,^^26^^,^^27^^,^^29^^,^^31^^,^^32^ Negative feelings, such as, pain, discomfort, anxiety, and low confidence in technology were considered as barriers.^8^^,^^15^^,^^26^^,^^30^

### Technology Design

In line with some of the subthemes on usefulness, specific findings emerged from multiple studies around the design of the technologies involved. The idea of a “steep learning curve” emerged again here; clinical use was considered to be hampered by having too-extensive instructions, a considerable need for support, technical problems, malfunctions, and specific constraints on the patient selection for use.^8^^,^^9^^,^^13^^,^^18^^,^^20^^,^^21^^,^^25^^,^^32^^,^^33^ Easy and intuitive set-up, don and doff, and maintenance were frequently stated requirements for rehabilitation technology.^14^^,^^15^^,^^27^ A lack of confidence in the technology impacted its potential use.^27^ There was a need for scores, other output data, and feedback from the device to be accurate at an appropriate level for the user and to be in line with the clinical treatment goals and decision-making,^8^^,^^12^^,^^13^^,^^15^^,^^27^ lending importance to the requirement for devices to be adjustable for a range of functional needs.^15^^,^^19^^,^^28^ Requirements on safety, including range of movement, pressure points on skin and cleaning, among others, were highlighted for robotic devices.^15^

### Social Interaction and Support

The role of clinicians was considered to be essential in the provision of support, mentorship, and motivation for people to use technologies, with clinicians acting as a conduit between the technology and the person using it.^17^^,^^19–21^^,^^24^^,^^33^ Carer and family support were of importance,^19^^,^^20^ although mixed perceptions were presented regarding the need for supervision by qualified staff.^15^ Regardless of the type of technology used, rehabilitation therapy using it needs to be meaningful, safe, effective, and efficient.^15^^,^^33^ A collegiate approach was considered to be important by clinicians; only 1 or 2 members of a team being involved was not seen to be sufficient to support its use in practice.^12^^,^^29^^,^^31^

### Integration With Current Service

Practical as well as contextual constraints arose when considering the integration of technologies within services. Practicality of use, size, and placement of technologies were important factors.^13^^,^^31^ Time for training in line with existing guidelines was a prerequisite for use, particularly considering the low-technology nature of current practice^28^^,^^29^^,^^33^as was the time for implementation, with 6 months considered being not long enough.^13^ An organizational culture with visible management support for technology use was considered to be of value.^9^^,^^14^^,^^24^^,^^27^^,^^28^ Further, a need for technology to align with clinicians’ existing professional roles and workplace environments was distinguished.^8^^,^^24^^,^^28^ High cognitive workload and unintentional burden on the therapist implementing or using the technology have been identified as the barriers for clinical uptake.^26^^,^^28^ Health care funding that allowed technological developments arose as a key influence on the uptake and use.^14^ The high cost and difficulty in persuading hospitals to invest in rehabilitation robots were considered to be the major barriers for implementation into clinical practice.^15^ Independent of the type of technology used, lack of skills, knowledge, and time were often identified as the barriers by the clinicians.^8^^,^^14^^,^^16^^,^^21^^,^^24^^,^^28^

### Improvements to Enhance Clinical Uptake

Several studies described a theme of suggested improvements to enhance clinical translation in general terms and specific to devices. Clinicians wanted to be involved and contribute to the development of technologies; they also wanted to get feedback on the previous developments and study findings.^31^ The evidence base was important.^14^^,^^18^^,^^24^^,^^29^^,^^33^ There may be a greater generalizability of use if the manufacturers broadened the criteria for patient selection for use.^12^ Specific to the device itself, subthemes here aligned with those above and included that technologies needed to be easy to use, durable, comfortable, low-risk, good value, and provide direct feedback.^14^^,^^28^ Further, they should not increase the clinicians’ workload and should align well with people’s rehabilitation goals and make a difference to everyday function.^13^^,^^18^^,^^24^^,^^31^

## Discussion

The primary finding is that reporting of sustained clinical uptake of technologies in neurorehabilitation practice is sparse. The situation is further compounded by the lack of clear definitions and language around the clinical implementation of technology in practice.

Despite the growing focus on the need to provide evidence of the uptake of technologies to enhance the delivery of care, only 16 of the 25 studies retrieved reported the actual amount of the use of technologies in clinical practice, and only 4 studies provided information on their sustained use. In general, while reporting of the actual uptake of technologies was somewhat limited, most studies focused on defining and finding solutions for the barriers to uptake such that we still do not have a clear picture of how much technology is actually being used in clinical or community therapy settings. This may, of course, relate to insufficient research funding to enable longer-term follow-up studies investigating technologies; indeed, Swank et al.^13^ found that the time needed for implementation was longer than anticipated. This lack of a clear picture on the technology uptake in neurorehabilitation presents a challenge—health technologies have the potential to meet the individual and service needs,^34^ but we still do not fully understand their pragmatic and sustained use in neurorehabilitation practice and how this might optimize the individual care and service delivery.

Our second objective helps to understand these challenges more fully in that it was to identify the factors reported to influence the uptake of technologies. Several barriers and facilitators were identified via this synthesis. One common barrier to adoption is the “steep learning curve” that needs to be overcome in order to engage with technology that may be complicated or require special training. Several solutions have been offered to address this, including the provision of adequate hands-on training^9^ and the availability of knowledgeable technicians and experts for in-clinic consultation and support^19^^,^^21^^,^^33^; this importance of the client–clinician relationship aligns with the previously published findings on supporting self-management with the use of technology.^3^ Indeed, clinicians have been described as potential technology “champions” for people with stroke.^35^ This problem might also be addressed by actively engaging users (ie, people with neurorehabilitation needs and clinicians) to advise the developers (ie, various types of engineers) in the codevelopment of devices.^5^^,^^35^ This would allow developers to recognize how to overcome some of the barriers experienced by clinicians and people with neurorehabilitation needs and to produce more user-friendly devices supporting people and services. However, the literature here suggests that the involvement of clients and therapists in the development of technology has been inconsistent. Involving users and therapists in the codesign and production of technologies from an early stage of development may be the first step in overcoming this barrier.

There was general agreement in this synthesis that clinicians and users should be involved in the decisions regarding the use of technology to enhance clinical uptake, which is in line with previous review findings on specific technologies such as robotics and virtual reality.^1^ To adopt technology, physical therapists should be part of a multidisciplinary team and should collaborate with other professionals to ensure that the technology matches their needs and the needs of their clients.^9^ People engaged in neurorehabilitation and their carers often have well-developed knowledge of technologies through self-education, and opportunities to enhance self-management are afforded.^2^ The role of the physical therapist is to ensure that the client is actively involved in decision-making and has considered all the possibilities when making a choice. This enables a personalized approach, where some of the concerns identified regarding the suitability of use in the light of challenges, such as possible fatigue, other medical needs, communication challenges, and meeting individual needs, can be considered.

In terms of perceived usefulness, there was general agreement that the use of technologies complements existing therapy by increasing treatment delivery, standardization of measurement of patient status and progress, and increasing the engagement of patients in their rehabilitation, enabling them to shape their goals according to their needs.^15^^,^^26^^,^^28^ There was optimism from both clinicians and people with neurological rehabilitation needs. Technology serves not to replace clinical therapy, but it is meant rather to expand the clinician’s toolkit and to enhance and personalize choices along a person’s rehabilitation pathway. Clinicians also expressed that perceived usefulness and benefit should be higher than the cost and effort of integrating technology into clinical practice^12^^,^^18^^,^^29^; this is an important consideration for often underresourced services. Indeed, high costs that prohibit investment from hospital services emerged as a major barrier,^15^ but the impact of an organizational culture with engaged management and clinicians who feel that the technology use aligns with their professional roles can improve the uptake and use. Such engaged service managers and clinicians can be powerful voices in influencing the future investment in technological advances for potential patient benefit.

### Study Limitations

The nature of a scoping review is such that there is a potential for bias, and methodological critique of included studies is not carried out. Further, our search strategy was necessarily broad to capture the relevant information on uptake, so elements of specificity may have been lost. No search of gray literature was undertaken at this scoping stage. However, the team mitigated for these limitations as far as was possible with a comprehensive and systematic approach, including the development of a search strategy in consultation with a faculty librarian in health sciences, team discussions at key decision points, and transparent reporting of searching, study retrieval, and inclusion decisions. The results also confirmed that the concept of “clinical uptake” was variously reported as such in the included studies. This in itself presented a possible limitation to searching for and reviewing the literature, as the concept of “uptake” is not easily defined. In future research, there is a need to have a clearer definition for determining and describing the uptake of technology in routine clinical practice.

## Conclusion

This scoping review of the literature exploring technology for neurorehabilitation has identified that reporting of clearly defined uptake and use in clinical practice are limited despite increasing recognition of the potential benefits afforded. Only 4 of 25 studies in our review provided the detail of sustained use of the technologies under investigation. However, the synthesis provided a comprehensive knowledge of barriers and facilitators to uptake. These included: an identified need for sufficient time to be allowed for the implementation of technologies in appropriate settings; recognition of a steep learning curve for clinicians and people with neurorehabilitation needs who can be helped by adequate training, clear instructions, clinicians supporting people to engage with technology and themselves being in a supportive organizational culture; and both clinicians and people with neurorehabilitation needs being involved in the design of devices and receiving feedback on the evaluation of those devices. We recommend that these important considerations are addressed in future protocols to provide an evidence-based, user-centered platform for the improved translation of technologies to neurorehabilitation clinical practice.

## Supplementary Material

2023-0076_R1_Suppl_Mat_I_pzad140

2023-0076_R1_Suppl_Mat_II_pzad140

## Data Availability

The authors confirm that the data supporting the findings of this study are available within the article and/or its supplementary materials.
