# Supplementary material for: Uptake of Technology for Neurorehabilitation in Clinical Practice: A Scoping Review
Source: Phys Ther. 2023 Oct 19;104(2):pzad140. doi: 10.1093/ptj/pzad140 (PMC10851848; doi:10.1093/ptj/pzad140)
Supplement: 2023-0076_R1_Suppl_Mat_I_pzad140 [file 2023-0076_r1_suppl_mat_i_pzad140.pdf]

Supplementary material I: Search strategy OVID MEDLINE

exp Neurological Rehabilitation/
neurorehab*.tw.
neuro* rehab*.tw.
(stroke adj2 rehab*).tw.
or/1-4
wearable electronic devices/ or fitness trackers/ or smart glasses/
exp virtual reality/
exp Video Games/
exp electronics/ or digital technology/ or electronics, medical/
wearable*.tw.
virtual reality.tw.
vr.tw.
gaming.tw.
video game*.tw.
gamifi*.tw.
robotic*.tw.
technolog*.ti,ab.
or/6-17
and/5,18
exp Adult/
exp Child/
21 not 20
19 not 22
limit 23 to "review articles"
23 not 24
limit 25 to yr="2000 -Current"
(uptake or implement* or translat*).tw.
26 and 27
