# Supplementary material for: Uptake of Technology for Neurorehabilitation in Clinical Practice: A Scoping Review
Source: Phys Ther. 2023 Oct 19;104(2):pzad140. doi: 10.1093/ptj/pzad140 (PMC10851848; doi:10.1093/ptj/pzad140)
Supplement: 2023-0076_R1_Suppl_Mat_II_pzad140 [file 2023-0076_r1_suppl_mat_ii_pzad140.pdf]

Supplementary material II- data extraction proforma

Supplementary material II- Data extraction

Study info.	Study id.
	Team member, data extraction
	Title
	Source
	Authors
	doi link
Population	n
	Users/patients, Health condition, Acute/Chronic
	Clinicians (Profession)
	Engineers
	Other
Type of study	Qualitative<br>(Method -Focus groups, Individual interview)
	Focus of qualitative evaluation<br>(Use time, adverse event, Barriers, facilitators, satisfaction, experience, perceptions, acceptability, feasibility, ease of use, uptake, usefulness, cost-effectiveness)
	Quantitative<br>(Method- Survey, Standardized questionnaire, survey, experimental)
	Focus of quantitative evaluation<br>(Use time, adverse event, Barriers, facilitators, satisfaction, experience, perceptions, acceptability, feasibility, ease of use, uptake, usefulness, cost-effectiveness)
	Mixed methods<br>(Method-Questionnaire/Experimental combination? Other?)
	Focus of Mixed methods<br>(Use time, adverse event, Barriers, facilitators, satisfaction, experience, perceptions, acceptability, feasibility, ease of use, uptake, usefulness, cost-effectiveness)
Type of technology	Robotics
	Neurophysiology
	Virtual Reality
	Exergaming
Technology maturity level	Commercial product
	Prototype evaluation
	Design development phase
	Prior design/production
	Technology readiness scale score (see TRL tab for details of scoring)

Supplementary material II- data extraction proforma

Evaluation Timepoint	Prior to implementation
	After implementation
	During implementation
	Before-after
	All stages
Intervention/Evaluation length	Single time point
	Pre-post
	Targeted use
	Actual use time
	Adverse events
	Adverse events-Reasons
	Missed sessions
	Drop outs
	Sustained use
	Pre-post-follow up (days/weeks/months)
Intervention provider & their profession	Supervised
	Unsupervised
	Semi-supervised
Phenomenon of interest	Off-the-shelf device
	User input in design
	Clinician input in design
	Barriers
	Facilitators
Application (findings relevant to)	Researcher
	Clinician
	User
	Other
Context/Setting	Research lab
	Home
	Community
	Online/Remote
	Clinic/hospital
	Urban
	Rural
	High income country + name
	Low and Middle Income country + name

Supplementary material II- data extraction proforma

Results	
